# Open-bigrams as orthographic processing units in Arabic: Evidence from the flanking-letters lexical-decision task

**DOI:** 10.3758/s13414-025-03061-2

**Published:** 2025-04-04

**Authors:** Hicham Zeghli, Christophe Cauchi, Mostafa Bouanani, Bernard Lété

**Affiliations:** 1 Laboratoire d’Études des Mécanismes Cognitifs, Université Lumière Lyon 2, Lyon, France; 2 Faculté des Lettres et des Sciences Humaines Dhar Elmehraz, Laboratoire SLLACHE, Université Sidi Mohammed Ben Abdellah, Fez, Morocco; 3 ATILF CNRS & Université de Lorraine, 54001 Nancy, France

**Keywords:** Orthographic processing, Arabic orthography, Open-bigram models, Flankers task

## Abstract

Using the “flanking-letters lexical decision” task, Dare and Shillcock *The Quarterly Journal of Experimental Psychology*, *66,* 487–504, (2013) and Grainger et al. *Acta Psychologica*, *146*, 35-40, (2014) demonstrated that word is facilitated when the flanking bigrams are present in the target word (e.g., RO ROCK CK), regardless of their position (e.g., CK ROCK RO), compared to different flanking bigrams (e.g., DA ROCK SH). This finding aligns with the Open Bigram Model proposed by Grainger and Van Heuven, (2004), which posits that orthographic representations in the Latin script are encoded by an unordered set of ordered letter bigrams. Employing the same task and experimental design, we replicated this key finding in Arabic. We observed a facilitative bigram-relatedness effect in both the repeated and the switched conditions. These results suggest that bigram coding reflects a universal orthographic mechanism, with letter bigrams functioning as representational units in Arabic, similar to their role in Latin scripts. Our findings also suggest that letter-position coding in Arabic may be more flexible than previously thought for Semitic scripts. We evaluate these conclusions within the framework of the Open Bigram Model and contrast them with the PONG model, which assumes absolute position coding.

## Introduction

Many studies on word recognition predominantly focus on the Latin script (used in languages such as English, French, and Spanish), which poses the risk of misapplying findings to other writing systems, including syllabaries (e.g., Japanese), logographies (e.g., Chinese), and abjads (e.g., Arabic). Therefore, it is crucial to determine whether significant effects observed in the Latin script can be replicated in other writing systems, and vice versa. By investigating both the similarities and the differences across writing systems, researchers can deepen their understanding of both universal and language-specific aspects of reading and orthographic processing. This serves as the primary objective of the current study, particularly in relation to orthographic processing, which involves the encoding of letter identities and positions during word recognition. This is assessed in Arabic through orthographic flanker effects in the Flanking-Letters Lexical Decision task (hereafter referred to as the FLLD task).

While the Arabic script has primarily been studied using masked priming tasks (see below subsection Orthographic processing in Arabic), the present study adopts the FLLD task as a novel approach to examining orthographic processing. Unlike masked priming, where stimuli are presented sequentially at the same spatiotopic location (a prime followed by a target), the FLLD task involves the simultaneous presentation of stimuli at distinct spatiotopic locations, with a target flanked by letters on both sides (e.g., RO ROCK CK). This distinction is critical because the FLLD task specifically targets the spatial distribution of orthographic processing, whereas the masked priming paradigm focuses on the early, automatic stages of word processing. Evidence supporting this important difference comes from Cauchi et al. (2020), who contrasted the two paradigms using the same stimuli to examine the role of phonological processing in visual word recognition. In the masked priming task, pseudohomophones (e.g., *roze* for *rose*) facilitated target word recognition. However, the same manipulation in the FLLD task did not produce significant differences between pseudohomophones and orthographic controls, suggesting that phonology does not contribute to flanker-related effects, which are instead driven primarily by orthographic overlap. Thus, the FLLD task offers a unique opportunity to assess the strength of abstract orthographic representations in a script characterized by distinctive visual features, such as ligatures and allographic variations.

### The Flanking-Letters Lexical Decision (FLLD) task

Dare and Shillcock (2013) reported a pivotal discovery in reading research using an adapted version of the flanker task by Eriksen (1995), the FLLD task. In this task, participants make lexical decisions regarding target words and nonwords flanked by bigrams positioned to the left and right of the target. They demonstrated that having identical flanking bigrams surrounding the target (e.g., RO ROCK CK) facilitated lexical decisions for word stimuli compared to conditions where flanking bigrams were absent from the target (e.g., DA ROCK SH). Importantly, this bigram-relatedness effect remained consistent regardless of the left–right ordering of the bigrams (RO ROCK CK = CK ROCK RO). Grainger et al. (2014) replicated this key finding and demonstrated that while bigram position does not influence the reading of French words, letter order does (RO ROCK CK ≠ OR ROCK KC).

About ten years after these seminal works, it is now well established that flanking letters influence the processing of central target words in the FLLD task, with both bigrams and words serving as flankers (see Grainger, 2024, for a synthesis of research in French within the open-bigram framework). For example, studies in French with word flankers have shown that, in adults, the integration of non-lexical information across spatially distinct repeated letter strings is primarily driven by orthographic information, with no role for phonological information (Cauchi et al., 2020). The FLLD task has also been used with children to examine the developmental trajectory of orthographic processing (Snell et al., 2021) in interaction with morphological information manipulated in word flankers (Cauchi et al., 2022).

These findings^1^ align well with the theoretical framework for multiple-word processing proposed by Mozer (1987), subsequently refined and extended by Grainger and van Heuven (2004), Grainger and Whitney (2004), Grainger and Ziegler (2011), and Grainger et al. (2014), detailing how orthographic representations are encoded as combinations of letter pairs. In their Open-Bigram Model, location-specific letter detectors operate in parallel across multiple words, signaling the presence of a given letter identity or inter-word space at a specific location relative to eye fixation. This information activates ordered pairs of contiguous and non-contiguous letter combinations, stored as an unordered set of “open bigrams.” These bigrams then activate whole-word orthographic representations, leading to unique word identification through a winner-take-all process. For a conceptually related model addressing letter-position representation and processing in visual word recognition, see the SERIOL model by Whitney (2001) and Whitney and Cornelissen (2008). For challenges to the role of open bigrams in orthographic processing, see, for example, Kinoshita and Norris (2013), Lupker et al. (2014), and the PONG model (Snell, 2024; Snell & Simon, 2025), which is discussed in the Discussion section in relation to the Open Bigram Model.

The Open-Bigram Model suggests that orthographic information extracted in parallel from both the fovea and parafovea jointly influences foveal word recognition. In this model, bigrams function as essential orthographic units in visual word recognition. For example, a word like “ROCK” activates bigrams representing adjacent letters (RO, OC, and CK) as well as non-adjacent ones (RC and OK). However, it remains unclear whether bigram flanker effects are specific to the Latin script and its associated writing systems (e.g., English, French, Spanish), or if they reflect a universal orthographic processing mechanism applicable across other scripts. If bigram coding is indeed a universal feature of orthographic processing, bigram flanker effects should also be observed in non-Latin writing systems.

To examine orthographic processing, the Arabic language offers a compelling contrast to the Latin script. Arabic utilizes an abjad script featuring letter ligatures and allographic variations and is read from right to left, making it inherently distinct from the left-to-right orientation of the Latin script.

### Orthographic processing in Arabic

Arabic is the official language in approximately 25 countries, spoken and read by nearly 400 million people. Its script has very specific characteristics and should be assessed for sensitivity to orthographic flanker effects in the same way as the Latin script. The Arabic orthographic system operates with an abjad alphabet of 28 letters that are written cursively from right to left (see Saiegh-Haddad & Henkin-Roitfarb, 2014, for a comprehensive review of Arabic orthography; and Bouanani, 2019; Bouanani & Rabie, 2015, for further details in Arabic). Most letters share the same base shape but differ with respect to the number and position of dots. For instance, the letter ب /b/ has a single dot underneath it, while ت /t/, and ث /θ/ have two and three dots above them, respectively. It is worth noting that the Arabic script is not exclusive to the Arabic language. It is also used for languages from other families, such as Persian, Urdu, and Uyghur. This highlights the adaptability of the script and its utility across diverse linguistic contexts and underscores the broader relevance of examining orthographic processing in the Arabic script across different languages.

A notable feature of Arabic orthography lies in its allographic nature: each letter can take up to four different forms depending on its position within a word (see Boudelaa et al., 2020, for frequency counts of Arabic letters and their allographs). These forms include the initial (عـ), medial (ـعـ), final (ـع), and isolated (ع) versions of the phoneme /ʕ/. Of the 28 letters, 22 are fully connecting; that is, they link to both preceding and following letters and thus have four allographs. The remaining six letters, however, are partially connecting, as they link only to the preceding letter and therefore have only two forms – an isolated and a final form (e.g., ر and ـر for the phoneme /r/).

An additional characteristic of Arabic orthography is its primarily consonantal structure. All letters represent consonant phonemes, with the exception of three letters that indicate the long vowels: ي for /iː/, و for /uː/, and ا for /aː/. The short vowels /a/, /u/, and /i/ have no corresponding letters but may be represented by diacritical marks above or below the consonants, especially in texts aimed at younger readers, such as elementary school reading materials.

Finally, Arabic has a unique two-dimensional structure, composed of a root and a pattern. The root typically consisted of three consonants, forming a triliteral sequence that encodes a general and abstract meaning. For example, the sequence کتب [**ktb**] means “that which relates to writing.” The formation of words in the Arabic lexicon requires the addition of a pattern that enables the derivation of various linguistics elements, including nouns, verbs, and adjectives. Thus, the nouns كاتِب [**k**a:**t**i**b**] (writer) and مكتب [ma**kt**a**b**] (office) are formed by combining the root كتب [**ktb**] with the noun patterns فاعِل [**C**a:**C**i**C**] and مفعل [ma**CC**a**C**], respectively (**C** = Consonant of the root).

Due to this unique characteristic, most studies on orthographic processing in Arabic have focused on word root effects. Research in Semitic languages has indicated that roots play a crucial role in word identification. Root-preserving primes facilitate word processing in both Arabic and Hebrew (Boudelaa & Marslen-Wilson, 2000, 2001, 2005; Frost et al., 1997, 2000). Similar priming effects have also been reported for root-preserving parafoveal previews in these languages (Deutsch et al., 2000, 2003; Hermena et al., 2021). However, such facilitation is eliminated if the order of root letters is not preserved (Hermena et al., 2021; Perea et al., 2010; Velan & Frost, 2007, 2009, 2011). This contrasts with European languages, where transposed-letter primes (e.g., *jugde* as a prime for the target *judge*) or parafoveal previews facilitate target-word processing relative to substituted-letter primes or previews (e.g., *junpe*–*judge*; Brysbaert, 2001; Dunabeitia et al., 2007; Johnson et al., 2007; Kinoshita & Norris, 2009; Perea & Carreiras, 2006a, b, 2008; Perea & Lupker, 2003). These findings suggest a rigid letter-position coding in Semitic languages and a more flexible letter-position coding in European languages (Frost, 2012, 2015). However, the nature of letter-position coding (rigid or flexible) may depend on the specific task employed. For example, Velan and Frost (2011) reported no root‐TL priming benefit for Hebrew target words in lexical decision. Conversely, Kinoshita et al. (2012) found a clear benefit from root‐TL primes in a word‐matching task using the same stimuli. In Arabic, Boudelaa et al. (2019) replicated the same pattern of task-dependent effects: root‐TL priming benefit was absent in lexical decision but was obtained in a matching task using the same stimuli. As argued by Hermena and Reichle (2020), the explanation for these findings is that, because lexical decisions entail lexical access, letter strings are influenced by the rigid letter‐position coding that supports morphological decomposition and processing. In contrast, the word‐matching task does not require lexical access and can be performed using pre‐lexical (abstract) letter codes as the basis of comparison, without being affected by the properties of the Arabic or Hebrew lexicons. We will return to these aspects of letter-position coding in the Discussion, contextualizing them both in light of our results and of the key features of the Arabic script.

### The present study

Investigating the orthographic processing of Arabic offers a unique lens through which to explore how the structural characteristics of different writing systems shape cognitive processes involved in reading. Arabic’s right-to-left script, its reliance on consonantal roots, and the presence of allographic variations and letter ligatures create a distinct orthographic landscape compared to Latin-based scripts, which are typically read from left to right and display less variability in letter forms. These differences allow researchers to examine how the brain adapts to diverse orthographic conventions and to identify both universal and script-specific mechanisms underlying reading.

To address these questions, we designed an experiment that directly compares the coding of bigrams in Arabic with that of Latin-letter words. The main objective of the study was to determine whether Arabic words are coded by open bigrams during orthographic processing in the same way as Latin-letter words. As in the experiments by Dare and Shillcock (2013) and Grainger et al. (2014), we employed the FLLD paradigm. A four-letter target word was presented with repeated bigrams (e.g., 43 4321 21, where the numbers represent the positions of letters), switched bigrams (e.g., 21 4321 43), or unrelated bigrams (e.g., 87 4321 65). The hypothesis was straightforward: if Arabic words are coded as an unordered set of bigrams, as proposed for the Latin script by Grainger and van Heuven (2004), recognition of the target word should be facilitated when flanking bigrams are orthographically related to the target word, regardless of their order (43 4321 21 or 21 4321 43), compared to when they are unrelated (87 4321 65).

## Methods

### Participants

One hundred and sixty participants were tested for the Experiment (102 boys, 58 girls; M_age_ = 22 years, SD = 6 years, 8 months). They were recruited from two locations: a group of 80 students from grades 10 (n = 43) and grade 11 (n = 37) from the Alkotrobi public high school in the city of Ouazzane (north-western of Morocco; M_age_ = 17 years, 1 month, SD = 1 year, 7 months), and a group of 80 bachelor (N = 25) and master (N = 55) students from the faculty of Letters and Human Sciences of Dhar-El-Mehrez in the city of Fez (north-western of Morocco; M_age_ = 26 years, 11 months, SD = 6 years, 3 months).

All the participants had normal or corrected-to-normal vision, were native to Morocco, and had learned to read the Arabic language in the first year of primary school.

### Design and stimuli

We employed four conditions (see Table 1). The first three conditions were adapted from the study by Grainger et al. (2014) who used bigram flankers in an Repeated condition (12 1234 34, corresponding to 43 4321 21 in Arabic), a Switched bigram flanker condition (34 1234 12, corresponding to 21 4321 43 in Arabic) and an Unrelated bigram flanker condition (56 1234 78 where 56+78 form another word, corresponding to 87 4321 65 in Arabic where 65+87 form another word 8765). The final condition was a No-flanker condition, allowing us to examine the impact of the mere presence of surrounding stimuli.

**Table 1:** Stimuli for the four experimental conditions expressed as letter numbers. Examples are provided for a word target [خيار] (*cucumber* in English) and a pseudoword [مائس] (ma’is in English transliteration)

Flanker conditions	Word target	Pseudoword target
No Flankers	4321 [خيار]	4321 [مائس]
Repeated Bigrams	43 4321 21 [خيرخيار ار]	43 4321 21 [ما مائس ئس]
Switched Bigrams	21 4321 43 [ار خيار خيـ]	21 4321 43 [ئس مائس ما]
Unrelated Bigrams	87 4321 65 [نغرخيار مة]	87 4321 65 [رو مائس تح]

*Note.* The two bigrams 65 and 87 form another word or pseudoword 8765

A set of 112 target words and 112 pseudowords were used, making a total of 224 experimental stimuli. A second set of 224 unrelated flanker items (112 words and 112 pseudowords) was paired with the first set for the purpose of the unrelated bigram condition (see Table 2 for statistics of the experimental material). Target and flanker words were selected from ARALEX (Boudelaa & Marslen-Wilson, 2010) and Perea et al. (2018). All target and flanker pseudowords were selected from Perea et al. (2018). To generate the unrelated bigram flankers, we ensured that none of the targets (words or pseudowords) were orthographically related to their flankers (i.e., no common letters). All stimuli are available in the Online Supplementary Material (see the *Data accessibility statement* section at the end of the paper for the link).

**Table 2:** Statistics of the experimental material

Item categories	N	Perea et al. (2018)/ARALEX)	Lexical frequency ^a^	Initial bigram frequency ^b^	Internal bigram frequency ^b^	Final bigram frequency ^b^
**Target words**	112	72 / 40	34 [0.03 – 275.3]	3.1	3.2	3.0
**Unrelated flanker words**	112	72 / 40	8.3 [0.03 – 200.7]	2.9	3.2	3.1
**Target pseudowords**	112	112 / 00	-	2.8	3.1	2.9
**Unrelated flanker pseudowords**	112	112 / 00	-	2.7	3.1	2.9

^a^ Mean frequency per million and range, from ARALEX

^b^ Mean log frequency per million, from ARALEX

Based on ARALEX, initial, internal, and final bigram frequencies were matched across the four item categories (target and flanker items for both words and pseudowords; see Table 2). The mean lexical frequency of target words was higher than that of unrelated flankers (34 vs. 8.3 per million words, respectively), but recall that unrelated flankers were presented only as bigrams, not as complete lexical units, with their bigram frequencies closely matched to those of target words.

Each stimulus presentation followed the same format: a central four-letter string with no repeated characters and no vowel diacritics, flanked on either side by a bigram. The visual form of the letters in the bigrams was maintained as it appeared in the target. We ensured that none of the targets formed a new word when combined with one of their bigram flankers, which could have otherwise biased lexical decisions.

### Procedure

The experiment was conducted on a 15-in. monitor with a resolution of 1,280 × 800 pixels and a refresh rate of 60 Hz. Stimuli were presented using DMDX (Forster & Forster, 2003). Each trial began with an 800-ms presentation of a fixation cross at the center of a dark screen. Then, the target, flanked by bigrams on each side (7° of visual angle), was displayed on the screen until a response was made or 3,000 ms had elapsed, in 17.5 pt Courier New font. The spacing between the target and its flankers corresponded to the width of a single character. Participants indicated as quickly and accurately as possible whether the target was a word or a pseudoword by pressing the M or Q button, respectively, on an AZERTY keyboard for right-handed participants. The reverse arrangement was used for left-handed participants. No feedback was provided after the responses. The participants were instructed to focus on the target in the center of the character strings and to ignore the adjacent characters.

To ensure that each participant saw each string only once, four versions of the experimental stimuli were created using a counterbalanced Latin square design, such that each target appeared in every experimental condition across the four versions but only once per condition in each version. There were 28 items per experimental condition (112 words or pseudowords divided across four experimental conditions).

The 224 trials were divided into eight blocks of 28 trials. A rest break was provided between each block. Both the order of the blocks and the trials within each block were randomized differently for each participant. The task began with 20 practice trials, followed by the main experiment. The experiment lasted approximately 15 min.

## Results

### Statistical power estimation

Only the data for words were analyzed. The dataset comprised 4,480 observations per condition (160 participants × 28 items), which significantly exceeds the recommended minimum of 1,600 observations recommended by Brysbaert and Stevens (2018).

Statistical power was further assessed through simulations with reduced samples to provide a conservative estimate. Specifically, the contrast between the No Flankers and Unrelated Flankers conditions within the Repeated condition was chosen to assess the power of what was anticipated to be the strongest effect. Statistical power was calculated using the Monte Carlo method through the *powerSim* function from the *simR* package (Version 1.0.5; Green & MacLeod, 2016). In each of the 20 iterations, the program randomly selected a subset of items and participants from the original dataset and fit a linear mixed-effects model to estimate statistical power. Each iteration retained 75% of the original sample size (i.e., 21 of 28 items and 120 of 160 participants). The resulting estimated statistical power was 0.93, demonstrating that the study had robust power.

All raw data and R analysis scripts are available in the Online Supplementary Material (see the *Data accessibility statement* section at the end of the paper for the link).

### Data trimming

The analyses of response times (RTs) excluded incorrectly answered trials (5.5%). Trials with RTs falling outside the range of 300–3,000 ms (0.05%) were also excluded. Subsequently, the RTs were log-transformed to meet normality assumptions. A null model with a random structure, including by-participant and by-item random intercepts, was employed to compute standardized residuals from the logarithmic RTs. Trials with standardized residuals greater than 2.5 in absolute value were excluded (2.6%).

The data were analyzed using the R statistical computing environment (R Core Team, 2018). For the RT analyses, linear mixed-effects models were fitted (Baayen et al., 2008) with the *lmer* function from the lme4 package (Version 1.1–21; Bates et al., 2019). For the error rate analyses, a generalized mixed-effects model was fitted using the *glmer* function from the same package, employing the same structure as the models used for RTs, with the error rate variable treated as a binomial response (1 for error, 0 for correct, with no intermediate values). The significance of the fixed effects was assessed using type III model comparisons with the *Anova* function from the car package (Version 3.0–8; Fox & Weisberg, 2011). Post hoc analyses were conducted using cell means coding and single degrees of freedom contrasts with the *glht* function from the multcomp package (Version 1.4–13; Hothorn et al., 2008), applying the normal distribution to evaluate significance. These analyses compared each condition with one another. Mean RTs and error rates per condition are presented in Table 3.

**Table 3:** Mean response times (RTs; in milliseconds) and errors rates (probabilities) for word targets (with standard deviations in parentheses) across the four flanker conditions

Flanker condition	RTs	Error rates
No flankers	751 (139)	.048 (.050)
Repeated bigrams	770 (143)	.049 (.056)
Switched bigrams	775 (146)	.057 (.062)
Unrelated bigrams	789 (143)	.064 (.076)

### Response time (RT) analyses

For the RT analyses, the main effect of Flanker Conditions was significant (χ2 (3) = 122.72, *p* < .001). In the post hoc comparisons, the No Flankers condition was significantly faster than the Repeated Bigrams condition (−19 ms, *z* = −5.17, *p* < .001), the Switched Bigrams condition (−24 ms, *z* = −5.90, *p* < .001), and the Unrelated Bigrams condition (−38 ms, *z* = −11.39, *p* < .001). Critically, both the Repeated Bigrams and Switched Bigrams conditions showed significantly faster RTs compared to the Unrelated Bigrams condition (−19 ms, *z* = −5.59, *p* < .001; −14 ms, *z* = −3.90, *p* < .001; respectively). Notably, there was no significant difference between the Repeated Bigrams and Switched Bigrams conditions (−5 ms, *z* = −1.48, *p* > .10). To further assess this comparison, we performed a Bayesian contrast to complete this last post hoc comparison using *lmBF* function from the BayesFactor package (Version 0.9.12-4.2; Morey, 2019). The BF_01_ revealed that the data were 13.60 more times likely to occur under the null hypothesis than the alternative hypothesis, providing positive evidence for the lack of difference between the two conditions.

### Error rate analyses

For the error rate analyses, the main effect of Flanker Conditions was significant (χ2 (3) = 12.14, *p* < .01). This effect was primarily driven by the Unrelated Bigrams condition, which resulted in a higher error rate than the three other conditions: an increase of 1.6% compared to the No Flankers condition (*z* = −2.95, *p* < .001), 1.5% compared to the Repeated Bigrams condition (*z* = −3.15, *p* < .001), and 0.7% compared to the Switched Bigrams condition (*z* = −2.41, *p* < .01). No other comparisons were statistically significant.

The analysis of error rates reveals a clear relationship between RTs and error rates, with no evidence of a speed-accuracy trade-off, as both RTs and error rates follow the same ranking. Participants achieved the fastest RTs in the No Flankers condition while maintaining low error rates, indicating that the absence of surrounding letters facilitated efficient information processing. Conversely, the slowest RTs in the Unrelated Flankers condition were accompanied by increased error rates, suggesting cognitive overload as participants processed letter strings in a manner akin to natural reading, where differing letters flank the fixated word. The Repeated and Switched conditions fell between these two boundaries.

## Discussion

The aim of this study was to investigate orthographic processing of written Arabic words using the FLLD task. We utilized a version where the central target word was flanked by bigrams on both sides, following Dare and Shillcock (2013) in English and Grainger et al. (2014) in French. These studies found that lexical decision responses to target words were facilitated when the flanking bigrams were part of the target word, compared to when they were not. This bigram-relatedness effect was observed regardless of the position of the bigrams, both in the repeated position (RO ROCK CK) and in the switched position (CK ROCK RO).

The bigram-relatedness effect is well explained by the Open Bigram model (Grainger & van Heuven, 2004; Grainger et al., 2014). According to this model, letter detectors that are sensitive to specific positions (retinotopic detectors) do not directly activate lexical representations. Instead, they pass information to an intermediate layer that encodes pairs of letters, or bigrams. These bigrams convey relative positional information, indicating whether one letter is to the left or right of another, rather than providing absolute positional data. At the bigram level, the model does not account for the exact location of letters in the visual field. Since word recognition is based on bigram detectors rather than precise letter positions, the model allows for a certain degree of positional flexibility.

Our study, conducted in Arabic, replicated these findings, marking the first time such results have been obtained in a non-Latin script. We observed a bigram-relatedness effect in both repeated and switched conditions, consistent with findings previously reported in English and French. Notably, participants achieved the fastest RTs in the No Flankers condition, suggesting that the presence of surrounding letters can impede reading, even when these letters overlap with those in the fixated word. Additionally, RTs in the Repeated Bigrams condition were comparable to those in the Switched Bigrams condition, indicating that the orthographic processing of the target word was not affected by bigram order.

Altogether, our results suggest that the cognitive mechanisms underlying orthographic processing in Arabic may be more similar to those of Latin scripts than previously thought (e.g., Frost, 2012, 2015), and that letter-position coding in Semitic languages could be as flexible as in European languages. This interpretation is consistent with studies demonstrating the presence of pure orthographic processing effects in Arabic, even in the absence of morphological overlap, as observed in masked priming tasks (e.g., Boudelaa et al., 2024; Perea et al. 2014). Such findings support the notion that the early stages of visual word recognition rely on universal orthographic mechanisms shared across languages, regardless of script-specific characteristics. The present study, conducted using the FLLD task, which thoroughly investigates the spatial distribution of orthographic processing, further reinforces this perspective by showing that bigram coding may function as an effective orthographic mechanism in visual word recognition, even in Arabic. Notably, these results were obtained in a script characterized by three key features – letter ligatures, allographic variations, and right-to-left reading direction – that could potentially modulate the bigram relatedness effect. We discuss these potential impacts below.

### Key features of Arabic script and bigram coding

To begin with, letter ligatures are an essential feature of Arabic script, serving both aesthetic and functional purposes. However, ligatures have been shown to complicate letter processing in Arabic by altering the visual appearance of individual letters and their combinations (e.g., Abu-Rabia, 2002; Taha & Ibrahim, 2013). Additionally, ligatures may distort the positional coding of letters, further complicating orthographic processing. These factors could increase cognitive load, slow word recognition, and reduce any bigram-relatedness effect between the fixated word and adjacent letters.

Indeed, previous research has highlighted the potential impact of ligatures on letter processing in Arabic script. For instance, Yakup et al. (2015), using a masked priming paradigm in Uyghur – an agglutinative Turkic language written in Arabic script – found that letter transpositions preserving ligature patterns facilitated word recognition, whereas transpositions disrupting these patterns were less effective. Conversely, Perea et al. (2013), also employing a masked priming paradigm, demonstrated that, despite the visual complexity of Arabic orthography, both adult and developing readers rapidly access abstract letter representations. This suggests that while ligatures may influence visual processing, their effects are mitigated by the efficient conversion of visual input into abstract orthographic representations.

Our results align with these conclusions. Despite the visual complexity introduced by ligatures, we observed evidence supporting the presence of open-bigram orthographic units in Arabic, similar to those found in Latin scripts. This indicates that Arabic readers can rely on open-bigram representations that facilitate flexible letter-position coding, even in the presence of ligatures.

Another notable feature of Arabic script is allographic variation, which refers to the different shapes a letter can assume based on its position within a word – initial, medial, final, or isolated. These variations may complicate orthographic processing in visual word recognition, as readers must recognize both the letter’s identity and its contextual form. For example, Boudelaa et al. (2019) investigated how allographic variation in Arabic script interacts with letter ligatures and the transposed-letter (TL) effect in visual word recognition. Their study found that, unlike in Latin scripts, TL priming effects do not occur during lexical access in Arabic, but strong TL priming effects were observed in a same-different matching task, significantly influenced by allographic variation.

In the present study, to better isolate the bigram-relatedness effect on lexical decision, as in Latin scripts, we maintained the shapes of the bigram letters as they appeared in the target, thereby avoiding allographic variations. In the Repeated Bigrams condition (43 4321 21) and in the Switched Bigrams condition (21 4321 43), where the flankers in both cases consisted of the letters of the target, potential allographic variations (i.e., instances where letters would typically undergo allographic changes) involved letter 2 of the right bigram (due to the following space) and letter 3 of the left bigram (due to the preceding space). These potential allographic variations affected 54% of the flankers (61 out of 112). In the Unrelated Bigrams condition (87 4321 65), where the flankers were constituted by another word, potential allographic variations involved letters 6 and 7, affecting 69% of the flankers (77 out of 112).

To further evaluate the potential influence of the absence of allographic variation in the flankers, we conducted a post-hoc analysis on reaction times by adding the variable "Allograph" to our factorial design. Each trial in the three flanker conditions (Repeated, Switched, and Unrelated) was categorized as involving the Presence or Absence of potential allographic variation. An ANOVA was then performed using a 2 Allograph × 3 Flanker Conditions design. The analysis revealed no significant main effect of the Allograph variable and no significant Allograph × Flanker Conditions interaction (both χ2 (2) < 1). This indicates that the absence of potential allographic variations in the flankers does not significantly influence the observed pattern of results. Overall, our findings support the notion of rapid access to abstract letter representations, which appears to be unaffected by the visual complexity of Arabic orthography, as previously concluded in the section above regarding the challenges posed by letter ligatures.

Remarkably, if the bigram-relatedness effect is also observed under conditions involving allographic variation, it would provide even stronger evidence for the involvement of abstract letter identity in this effect. A follow-up experiment could explore this hypothesis by creating four conditions: two orthographically related conditions between the target and the bigram flankers, with or without allographic variation of the letters (as in the present study), and two orthographically unrelated control conditions, again with or without allographic variation. Additionally, four conditions manipulating inverted flankers could be introduced.

Such an approach could further clarify the role of abstract letter representations in the flanker relatedness effect. Notably, this effect may be more easily demonstrated in Arabic script, where allographic variation exists, than in Latin script, where it does not.

A final point we address concerns the right-to-left reading direction of Arabic and its potential impact on the flanker-relatedness effect. The interaction between reading direction and letter-position coding in orthographic processing is an important area of study, particularly for understanding how right-to-left scripts influence reading patterns, visual perception, and cognitive processing. Research on natural reading in Arabic has shown that right-to-left orientation requires specific adjustments in visual processing strategies, leading to unique eye movement and fixation patterns distinct from those observed in left-to-right languages (e.g., AlJassmi et al., 2022; Almabruk et al., 2011).

In ongoing research, we have collected data from Arabic readers to explore a potential leftward bias in the FLLD task. The data are currently being analyzed, and we anticipate presenting a comprehensive manuscript with our findings in the near future. This question was recently examined within the French script by Cauchi et al. (2024), who investigated rightward bias in children from grades 1 to 5 using the FLLD task, with an additional adult group for comparison. The task presented a central target word flanked by either identical or different words on the left (e.g., park park #### / five park ####), the right (e.g., #### park park / #### park five), or both sides (e.g., park park park/five park five). Results showed a greater increase in flanker-relatedness effects from Cycle 2 to Cycle 3 in both the bilateral and right-sided conditions, as compared to the left-sided condition. This pattern suggests that rightward bias arises from attentional asymmetries developed through the process of learning to read, rather than from an innate hemispheric specialization for linguistic processing. These findings imply that the rightward bias in attention allocation in left-to-right scripts may have an equivalent, but reversed, counterpart in Arabic reading.

These analyses of key features of the Arabic script challenge the assumption that script complexity necessarily leads to greater positional uncertainty. Instead, the presence of ligatures and allographic variations does not seem to disrupt letter-position encoding; on the contrary, it may contribute to a more robust form of orthographic processing. Overall, our results provide strong evidence that Arabic readers process bigrams as flexibly as, if not more than, Latin script readers. Specifically, we observed a bigram-relatedness effect in both the repeated and switched conditions, with no significant difference between them, supporting the relative position encoding scheme of the Open Bigram Model.

Consequently, our results have important implications for models of orthographic processing in the FLLD task. In the following section, we discuss the PONG (Position-Ordered Neural Grouping) model (Snell, 2024; Snell & Simon, 2025), a recent alternative to the Open Bigram Model. Since the switched condition effect is central to the debate on letter position coding, we contrast PONG’s absolute position encoding with the relative position encoding proposed by the Open Bigram Model. Given that PONG has been explicitly tested in the FLLD task, it provides a particularly relevant framework for discussing our results in Arabic script.

### Implications for models of orthographic processing

The PONG model, developed by Snell (2024) and expanded by Snell and Simon (2025), is a neural framework for encoding the spatial positions of visual stimuli. It has been applied to reading and attentional control and has been tested with the FLLD task to assess how position-based encoding influences cognitive processing. At its core, PONG assumes absolute position encoding, each letter being encoded in a fixed spatial location, forming stable neural representations that dynamically adapt to context. In the FLLD task, the switched condition is a critical test of PONG’s absolute encoding hypothesis. According to Snell and Simon (2025), this positional shift violates absolute position expectations, leading to a significant decrease in the orthographic relatedness effect.

Analyses of the switched condition in Latin script further inform the debate on absolute vs. relative letter position encoding (Snell & Simon, 2025). While equal facilitation from switched flankers strongly supports relative position coding, previous findings are not entirely univocal. Studies using four-letter targets with two-letter flankers (e.g., Dare & Shillcock, 2013; Grainger et al., 2014) have consistently reported no significant difference between the repeated and switched conditions, reinforcing the Open Bigram Model’s assumption that letter identity matters more than exact position. However, Snell and Simon (2025) questioned the statistical power of these studies, suggesting that the non-significant difference between the switched and repeated conditions may result from insufficient sample sizes, potentially leading to Type II errors. Moreover, Snell et al. (2018) introduced a key variation: when using six-letter targets with three-letter flankers, they observed that switching flanker positions slightly reduced facilitation.

Overall, these discrepancies raise an important theoretical question. If absolute position coding were the dominant mechanism, flankers should only facilitate word recognition when appearing in their original positions – yet this is not observed in most studies. Conversely, if relative position coding were the dominant mechanism, stimulus length should not influence the effect of switching flankers – yet Snell et al. (2018) found that larger positional shifts matter more for longer words.

Returning to PONG, Snell and Simon (2025) argue that it accounts for these discrepancies by encoding absolute letter positions while still allowing for some positional uncertainty, as proposed in Split Fovea Theory (Brysbaert, 2004; Ellis, 2004). This class of models suggests that while letter positions are encoded with some degree of flexibility, the visual system retains a coarse-grained spatial representation of letter order, which may become more relevant as word length increases.

However, our findings in Arabic suggest that position invariance remains a dominant factor in bigram encoding, aligning more closely with the Open Bigram Model than with PONG’s absolute position account. Notably, RTs in the Repeated Bigrams condition were comparable to those in the Switched Bigrams condition (tested by Bayesian analysis), indicating that orthographic processing of the target word was not affected by bigram order. Furthermore, our study was designed to ensure robust statistical power, addressing concerns raised by Snell and Simon (2025) about prior studies. With a large dataset and high estimated power, our findings are highly reliable. This methodological strength reinforces our conclusion that Arabic word recognition, like Latin script, follows a relative position coding mechanism, aligning with the Open Bigram Model rather than PONG.

## Conclusion

Our results suggest that bigram encoding in Arabic closely parallels that of Latin scripts, reinforcing the idea that orthographic processing operates on universal principles that transcend script-specific characteristics. These findings contribute to growing evidence that abstract orthographic representations play a central role in visual-word recognition across writing systems, even in scripts with distinctive features such as ligatures and allographic variations.

Grainger and van Heuven’s (2004) Open Bigram Model posits that visual word processing involves non-contiguous letter bigrams, allowing for greater positional flexibility. In languages like Arabic, which feature ligatures, allographic variations, and a right-to-left reading direction, the robustness of open bigrams may stem from their ability to capture abstract relationships between letters beyond specific spatial positions.

As our results support, this abstraction could be a universal feature of word recognition, enabling orthographic processing across diverse scripts despite their visual and directional differences. However, further research is needed to confirm whether universal cognitive mechanisms underlie visual-word recognition across different writing systems, from alphabetic scripts like Latin and Cyrillic to logographic scripts like Chinese.

## Data Availability

All stimuli used in this study, along with raw data and R analysis scripts, are available on the Open Science Framework repository at: https://osf.io/2pkbd/.

## Footnotes

1 Note that, in order to best address our main objective – investigating for the first time whether Arabic script can produce flanker effects – we concentrate this paper on the bigram-relatedness effect, as is elaborated upon in the following sections.
